# Does endoscopic sinus surgery alter the biomechanics of the orbit?

**DOI:** 10.1186/s40463-020-00442-5

**Published:** 2020-06-26

**Authors:** Leigh J Sowerby, Matthew S. Harris, Rootu Joshi, Marjorie Johnson, Tom Jenkyn, Corey C. Moore

**Affiliations:** 1 Department of Otolaryngology – Head & Neck Surgery, Schulich School of Medicine & Dentistry, Western University, London, Ontario Canada; 2 Department of Otolaryngology – Head & Neck Surgery, Division of Facial Plastic & Reconstructive Surgery, Schulich School of Medicine & Dentistry, Western University, London, Ontario Canada; 3 Department of Anatomy and Cell Biology, Schulich School of Medicine & Dentistry, Western University, London, Ontario Canada; 4 Department of Mechanical and Materials Engineering, Western University, London, Ontario Canada

**Keywords:** FESS, ESS, Sinus surgery, Orbit fractures, Ethmoid bulla, Lamina papyracea, Orbital trauma, Facial buttress, Medial orbital wall

## Abstract

**Objective:**

The purpose of this study is to determine if removal of ethmoid cell septations as commonly performed in endoscopic sinus surgery leads to a change in orbital wall fracture patterns and the force required to create them.

**Methods:**

Six fresh-frozen cadaveric heads were acquired and underwent endoscopic uncinectomy, maxillary antrostomy, and anterior and posterior ethmoidectomy on one, randomized, side. The contralateral sinuses were used as intra-specimen control. Hyaluronic acid gel globe injections were performed to simulate normal intra-ocular pressure. Post-op CT scans confirmed no orbital fractures or violation of the lamina papyracea prior to trauma testing. Orbital trauma was induced using a guided weight-drop technique. Both orbits were tested in random order, and sequentially higher drops were performed until both the test and control side demonstrated an orbital fracture on CT scan.

**Results:**

In all six heads, the post-sinus surgery side incurred a medial orbital wall fracture, and no orbital floor fractures were identified. On the other hand, on the control side, all six heads incurred orbital floor fractures at drop heights equal to, or higher than, the surgical side. Fisher’s exact test demonstrated a significant difference in fracture pattern (*p* <  0.001).

**Conclusions:**

To our knowledge, this is the first demonstration that the structures removed during sinus surgery may act as a buttress for the medial orbital wall. The anatomic changes of sinus surgery may alter the biomechanics of the orbit and affect the pattern of subsequent traumatic blowout fractures.

## Introduction

The normal functions of the paranasal sinuses have long been postulated and thought to be many. Theoretical functions include decreasing the weight of the skull, increasing the surface area of the nasal mucosa, enhancing resonance for speech and the production of nitric oxide [1]. The sinuses have also been shown to act as a “crumple zone” to protect the eye during maxillofacial trauma [2]. In the hydraulic theory of orbital fractures, when a force is applied directly to the globe, hydraulic pressure is transmitted through the globe, and an orbital blowout fracture into a sinus occurs. Despite its thin nature, the medial orbital wall, or lamina papyracea, is much less likely to fracture than the orbital floor. One review of orbital trauma demonstrated an 84.2% rate of isolated orbital floor fracture, compared to a 0.2% rate of isolated medial wall fracture [3].

Rationale for this difference in fracture pattern has been postulated to be that the uncinate process and ethmoid air cells act as a buttress for the medial orbital wall, protecting it from fracture [3]. The concept of the facial buttress is not new. In 1901, Rene Le Fort published his seminal work and classification scale [4]. His experimental studies involved applying blunt force to cadaver heads and observing the fracture patterns. This led to detailed understanding of the horizontal and vertical buttresses of the facial skeleton, but a similar investigation of buttresses of the medial wall has not been done.

A small case series reported medialization of the lamina on the order of 2-5 mm post-ESS [5]. Endoscopic sinus surgery may alter the natural buttress of the medial orbital wall and could lead to an increased risk of medial orbital wall fracture in the post-ESS patient who subsequently incurs maxillofacial trauma.

Therefore, the purpose of this experimental cadaver study was to determine if endoscopic sinus surgery leads to a change in the pattern of orbital blowout fractures and a reduction in the force required to create them.

## Methods

Six fresh cadaveric human heads were harvested from specimens in the Human Anatomy Lab at Western University, London, Ontario. All data was obtained in accordance with the Anatomy Act of Ontario and Western’s Committee for Cadaveric Use in Research, REB #06232015. The specimens were removed from fresh, non-perfused cadavers and subsequently frozen. Twenty-four hours before each procedure, the heads were defrosted to room temperature. The mean age was 79.5 ± 17.1 (age range 41–98) years with the study population consisting of 3 males and 3 females.

**Fig. 1:**
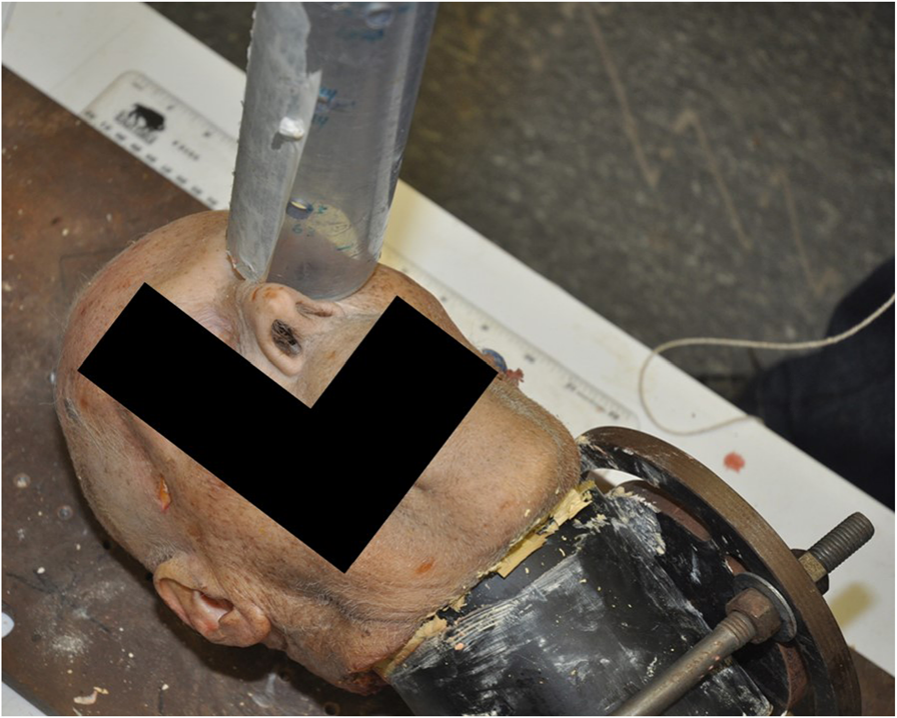
Immobilization of the heads in cement filled PVC pipe fastened to an iron bracket

A similar protocol to that described by Kellman and Schmidt was used to recreate hydraulic mechanism of trauma to the orbit [2]. Each head was potted in a 4″ PVC pipe filled with dental cement (Denstone Golden, Modern Materials, IN, USA), and mounted in an iron bracket for immobilization during testing (Fig. 1). Endoscopic uncinectomy, maxillary antrostomy, anterior and posterior ethmoidectomy was performed on one, randomized, side of each head by a fellowship-trained Rhinologist. A frontal sinusotomy was not performed. Uncinectomy was performed using a swing-door technique with a pediatric back-biter, and ethmoidectomy was performed with a J-curette and thru-cutting instrumentation to skeletonize the lamina. The un-dissected side was used as an intra-specimen control. Pre- and post-operative axial, coronal and sagittal reconstruction CT scans were performed on all fresh-frozen heads to confirm no orbital fractures or violation of the lamina was present prior to trauma testing. The same CT scanner was used to obtain all CT scans using 0.5 mm axial cuts reformatted to sagittal and coronal slices using the standard protocol for CT facial bones at our institution. 3D reconstructions were also performed. CTs were reviewed by a senior Otolaryngology resident and faculty member independently to confirm the presence or absence of a fracture sequentially during the trial. Where results were discordant, scan images were reviewed together, and a consensus was reached.

Hyaluronic acid gel globe injections were performed on all fresh-frozen heads for restoration of intraocular pressure to the normal value of 15 mmHg. Between 2.5 and 4 mL of sodium chondroitin sulfate (40 mg/mL) - sodium hyaluronate (16.5 mg/mL) gel (DisCoVisc, Alcon, TX, USA) was injected into each globe using a 27-gauge syringe. Intraocular pressure was confirmed through measurement with a Schiotz tonometer (Sklar Surgical Instruments, PA, USA).

A guided weight-drop methodology was constructed consisting of a vertical plastic tube with markings at 0.2 m increments to measure drop height. A 1.35 kg weight was used to deliver the impact force to the orbits. A tapered ‘nose’ was attached to the bottom of the weight to provide an impact area of 767 mm^2^ to mimic impact from a spherical projectile. The weight was fitted with a threaded cord allowing elevation of the weight within the tube. Positioning the tapered weight over the orbit allowed accurate targeting of the globe.

Drop testing began with the impact device at a height of 0.46 m for each fresh-frozen specimen based on previous studies [2]. A pre-experimental decision was made to use enough force to potentially cause a fracture, due to the risk of weakening the orbital bones from repeated trauma. Energy delivered by the impact device was calculated by measuring the gravitational potential energy of the system: E_p_ = mgh, where E_p_ = energy (J), m = mass (kg), g = 9.81 m/s^2^, and h = height (m). Selection of the first side for impact (control side or surgical side) was randomized for each head, using an online randomization tool. After delivering one strike to each orbit, the heads underwent CT scanning to determine if fractures had occurred. After each drop, the IOP was re-tested and ensured to be 15 mmHg. The procedure was repeated by dropping the weight from progressively higher heights until there was radiographic evidence of orbital fracture on both the test and control side. Once a fracture occurred on one side, drops were repeated on the contralateral side until a fracture was induced.

Statistical analysis was performed using Mann-Whitney U test for comparison of non-parametric ordinal data. Fisher’s exact test was used to compare categorical variables. All analysis was performed using SPSS Statistics for Windows (Version 20.0. Armonk, NY).

## Results

**Fig. 2:**
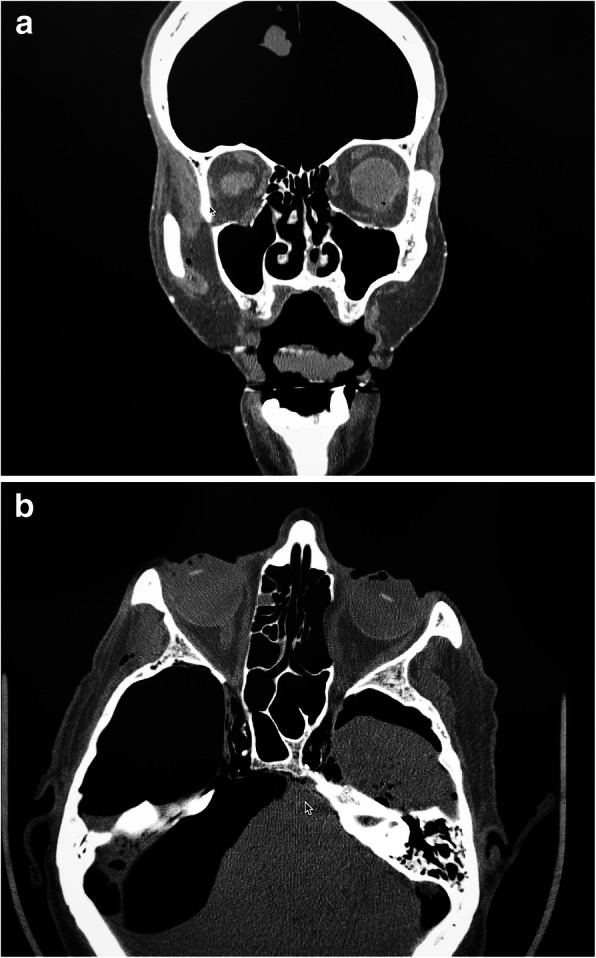
**a** Representative coronal CT. Note the intact ethmoid air cells and orbital floor fracture on the specimen’s right. On the left, post-FESS changes and a medial orbital wall fracture are seen. **b** Axial view of the same specimen, again demonstrating intact lamina on the right, post-FESS changes and a medial wall fracture on the left

In all 6 heads, the post-ESS sides incurred a medial orbital wall fracture. No orbital floor fractures were identified. On the control side, all 6 heads incurred orbital floor fractures, and no medial wall fractures were seen (Fig. 2). In all cases, the fractures seen were quite obvious when present and no discrepancy was seen between the two surgeons reviewing the CT scans. In seven of the 12 sides, a fracture resulted after the first weight drop. Fisher’s exact test demonstrated a significant difference in fracture pattern between post-FESS and control sides (*p* <  0.001). Mean energy required for fracture was 6.29 ± 0.43 J on the post-FESS side and 6.55 ± 0.40 J on the control side, without statistical significance (*p* = 0.23). Results are summarized in the Table 1.

**Table 1:** Summary of Fracture Results, Patterns and Energy Required

Cadaver	Post-FESS			Control		
	Impact Energy (J)	Medial Wall	Orbital Floor	Impact Energy (J)	Medial Wall	Orbital Floor
1	6.11	✔	✗	7.17	✗	✔
2	6.11	✔	✗	6.64	✗	✔
3	6.11	✔	✗	6.11	✗	✔
4	7.17	✔	✗	6.11	✗	✔
5	6.11	✔	✗	6.64	✗	✔
6	6.11	✔	✗	6.64	✗	✔
	Mean (SD)	Total	Total	Mean (SD)	Total	Total
	6.29 (0.43)	^a^6	0	6.55 (0.4)	0	^a^6

^a^Fisher’s Exact Test p < 0.001 (Post-FESS x Control)

## Discussion

This study provides the experimental evidence to support the hypothesis that the sinuses have evolved to carry out their respiratory function while providing buttress support to the medial orbital wall. A recent review of orbital fractures by Choi et al [6] supports our results. They identified a paradoxical predominance of medial wall fractures in their Korean patient population. In cases of isolated medial wall fractures, they noted a statistically significant decrease in the number of ethmoid air cell septations. This directly supports our findings, as the post-ethmoidectomy state is essentially the most extreme example of decreased ethmoid septations.

There are two accepted mechanisms for orbital floor fractures. The hydraulic theory suggests that force is transmitted through the globe and fractures an orbital wall via a transmission of energy. This preferentially happens in the orbital floor, likely due to the external buttress that the ethmoid air cells apply to the lamina. In the buckling theory, a force is transmitted through the inferior orbital rim and maxilla, causing a direct fracture to the orbital floor. This likely partially accounts for the increased number of floor fractures compared to medial wall fractures in retrospective reviews. In order to study only hydraulic force fractures, we excluded two heads with deep enophthalmos, as the impact device was unable to avoid the inferior orbital rim and provide a force directly to the globe, leaving us with 6 specimens for experimental study.

The variability of the superior attachment of the uncinate process is well described [7]. Certainly, the amount of support it applies to the medial orbital wall would vary depending on whether it attaches to lamina, skull base or middle turbinate. Although there did appear to be lamina attachment of the uncinate in all the specimens we tested, this study focused on the post-ESS surgical changes that include complete ethmoidectomy. As such, these results should be applicable to any skeletonized lamina, regardless of pre-operative bony configurations. It may be possible that the ethmoid septations, bulla, and middle turbinate lamella play a much more significant role than the uncinate process in buttressing the medial orbital wall. Cunnane et al demonstrated medialization of the lamina by 1-5 mm on CT in a small case series post-ESS, lending additional support to the buttress effect of the non-operated middle meatus [5].

The Mann-Whitney U-test was used to compare differences between groups, as a calculation for normalcy was not possible given the small sample size. The initial drop-test from 0.46 m produced an impact energy of 6.11 joules. This starting point was chosen based on previous work our group has done with cadaveric models. Five out of the 6 surgical sides fractured with the initial drop, while only 2 of the 6 control sides did. It is possible that a greater difference between mean energies would have been identified had a lower initial impact energy used. Unfortunately, with the limited access to the CT scanner for cadaveric work, we proceeded with the initial drop test on all specimens at the same time, thus not allowing us to adjust the force used. Ramesh et al. performed a similar study to the one presented here and found almost 50% less force was required to cause an orbital fracture after ethmoidectomy. They also found that all specimens with an ethmoidectomy had medial fractures versus 20% in controls [8].

Some limitations of this study warrant discussion. There was a limited number of heads available for testing; despite this, there was significance in the Fisher’s Exact Test. Second, the average age of the specimens used was almost 80. There is a possibility that the findings in these models may not be generalizable to the population as a whole, but the authors have not felt that to be the case during course cadaveric dissections in the past. Third, the cadaveric model is non-living and, therefore, not able to heal after surgery. It is possible that there were micro-fractures of the lamina that, although not detected clinically or on CT, predispose it to fracture. Therefore, these results are most applicable to the immediate post-operative period. However, the Choi et al review does support the concept that the ethmoid air cell septations are playing a role in supporting the lamina even in the virgin nose. We used the contralateral orbit in order to control for inter-specimen variables such as race, age, and bone mineral density. It is, however, one complete facial skeleton, and there may be an effect on the contralateral side during testing. In order to minimize this, we were blinded to the surgical side during trauma testing and randomized which side was hit first for every round. This is in keeping with similar methodologies in the literature [2]. As all heads were of Caucasian ethnicity, these results may not be generalizable to other ethnicities. Lastly, with this study, we were unable to tease apart the role that the various anatomic structures play in isolation to as a buttress but rather can only comment as a whole on the effect of removal of the ethmoid septae, ethmoid bulla and uncinate. We also did not include dissection of the frontal recess, the air cells of which may well serve to further buttress the orbit.

An increased risk of medial wall fracture has clinical significance. In a recent review, *Andrews* et al note that medial wall fractures have a two-fold increase risk of ocular injury compared to floor fractures [9]. This includes globe injury, vision loss and long-term diplopia. Medial wall fractures also require a more complex surgical reduction, often via a trans-caruncular approach. Although the rate of orbital trauma in the post-ESS patient is likely quite low and unpredictable, it behooves us as surgeons to understand what effects surgical procedures may have on our patients. Perhaps there is a certain subset of patients, such as martial arts practitioners, racquetball players or contact sport enthusiasts, who would reasonably consider an increased risk of complicated orbital fracture prior to consenting for a sinus procedure. This study may strengthen the argument for tools such as balloon sinuplasty in specific cases that aim to treat functional sinus obstruction, while maintaining as much normal anatomy as possible. At the very least, this study identifies an important relationship between the ethmoid air cells and the lamina. Understanding this will allow sinus surgeons to counsel patients appropriately should the need arise.

## Conclusion

In conclusion, this study provides evidence that the ethmoid bulla, middle turbinate lamella and/or uncinate may act as a buttress for the medial orbital wall. Endoscopic sinus surgery may alter this biomechanical relationship and affect the pattern of subsequent orbital fractures in the post-ESS patient.

## Data Availability

All data generated or analyzed during this study are included in this published article.
